# Human-animal interaction and One Health: establishment and validation of the Brazilian version of the Animal Empathy Scale

**DOI:** 10.31744/einstein_journal/2024AO0685

**Published:** 2024-09-09

**Authors:** Roberta Maria Savieto, Lucas Pires Garcia Oliveira, Gustavo Benvenutti Borba, Elivane da Silva Victor, Sabrina Bortolossi Bomfim, Letícia Bernardes de Oliveira, Giulia Catissi, Karina Pavão Patrício, Edgard Joseph Kiriyama, Eliseth Ribeiro Leão

**Affiliations:** 1 Hospital Israelita Albert Einstein Education and Research Center São Paulo SP Brazil Education and Research Center, Hospital Israelita Albert Einstein, São Paulo, SP, Brazil.; 2 Universidade Tecnológica Federal do Paraná Graduate School on Biomedical Engineering Department of Electronics-DAELN Curitiba PR Brazil Department of Electronics-DAELN, Graduate School on Biomedical Engineering, Universidade Tecnológica Federal do Paraná, Curitiba, PR, Brazil.; 3 Hospital Israelita Albert Einstein São Paulo SP Brazil Hospital Israelita Albert Einstein, São Paulo, SP, Brazil.; 4 Universidade de São Paulo Botucatu SP Brazil Universidade de São Paulo, Botucatu, SP, Brazil.

**Keywords:** Empathy, Animals, Human-animal interaction, One health, Factor analysis, statistical, Surveys and questionnaires

## Abstract

**Objective:**

To design and validate a Brazilian version of the Animal Empathy Scale, based on the existing Portuguese version.

**Methods:**

Content validity assessment was performed by expert judges, and the adapted scale was administered to a sample of 386 participants. Exploratory and confirmatory factor analyses were performed.

**Results:**

The bifactorial profile of the scale remained consistent, comprising Empathic Concern for Animals (Cronbach's alpha and McDonald's omega coefficients: 0.75) and Emotional Attachment with Animals (Cronbach's alpha and McDonald's omega coefficients: 0.79). Considering the One Health framework, collaborative, multidisciplinary, and intersectoral approaches are essential for achieving optimal health conditions for people, animals, and the environment given their intricate interconnections. Empathy plays a crucial role in promoting proximity between humans and animals, fostering positive connections that encourage biodiversity conservation.

**Conclusion:**

The 13 statements were retained, confirming the validity of the animal empathy scale for use in Brazil, and a Brazilian version of the Animal Empathy Scale was established.

## In Brief

In this study, we adapted and validated the Animal Empathy Scale for Brazil using 386 respondents based on psychometric steps. The two subscales-Empathic Concern with Animals and Emotional Attachment with Animals-were maintained with good internal consistency (evidenced by Cronbach α and McDonald Ω). We configured the only national instrument for this purpose, the Brazilian version of the Animal Empathy Scale.

## Highlights

From the One Health approach, empathy represents the initial movement towards achieving planet harmony.
The more emotions we attribute to animals, the more we develop pro-conservation behaviors.
AES-Brazil may serve as a tool for reflection and monitoring interventions in human-animal relationships.

## Graphical abstract

**Figure:**
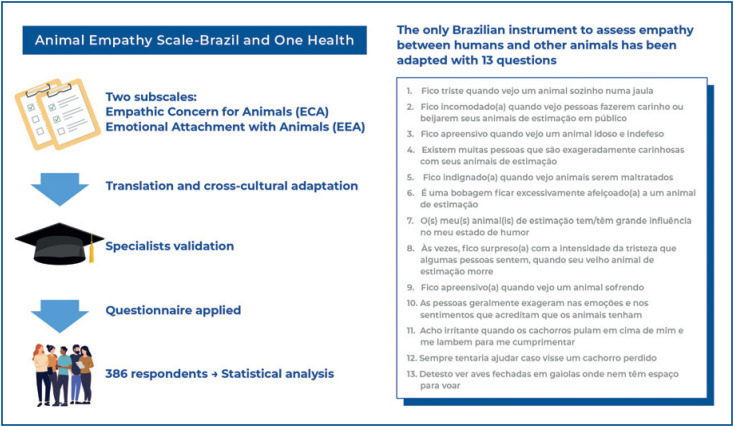

## INTRODUCTION

The interplay between the environment and health has been long recognized. However, in recent decades, with the rise in the incidence of zoonotic diseases, such as COVID-19, the concept of One Health has increasingly gained attention. This approach emphasizes the integration of strategies to address emerging, reemerging, and prospective diseases.^(1,2)^

The Manhattan Principles, published in 2004 following the "One World, One Health" meeting convened by the Wildlife Conservation Society, introduced significant objectives aimed at combating threats to life on Earth. These principles recognize the intricate relationship between human and animal health, encompassing both positive and negative aspects.^(3)^

One Health represents a transdisciplinary approach aimed at reorganizing human coexistence with the earth, encompassing all forms of life and ecosystems. The primary objective is to improve health outcomes for all species.^(4)^ This approach also allows comprehending the intricate relationships between humans, animals, and the environment, prompting reflection on how we can enhance our awareness and support for other species to foster a sense of harmonious interdependence.^(5)^ One Health emphasizes the need for encompassing the health and sustainability of the world's ecosystems, acknowledging the profound influence of the environment on human and animal health. This influence is extended through various factors, including resource availability (such as food and water supply), global climate, and air quality.^(6)^

The significance of the interactions between humans and animals extends beyond the emergence of new zoonotic diseases. It is crucial to recognize the positive aspects of this relationship. Empathic exchanges between humans and other animals, facilitated by connections with nature, play an essential role in promoting health and well-being.^(7,8)^

Within the One Health framework, *empathy* emerges as a critical tool for recognizing the needs of diverse living beings. Despite receiving considerable attention in recent years, the concept of empathy remains a subject of ongoing debate and lacks consensus.^(9-11)^ However, it consistently involves understanding the circumstances of others, encompassing affective and cognitive dimensions.^(11)^

Following current discussions, in this work, we consider empathy (directed towards any animal, whether human or not) as comprising three fundamental pillars: *affective*, which refers to the ability to feel the situation of others, *cognitive* which represents the ability to understand what a given situation means to the other, and *behavioral* which implies expressing such understanding in the sense of relieving/assisting others.^(12-14)^

Worldwide, health professionals dedicate several publications on the subject, as direct engagement with individuals and the need for interaction extends beyond immediate care raise doubts, demands, and solutions in the sphere of interpersonal relationships.^(15)^

In fields involving engagement with animals, such as biology and veterinary science, there has been discussion and refinement of the transition from inter-human empathic behavior to inter-species empathy. This transition is prompted by acknowledging that our interactions with other living beings subject us to their perceptions and understanding of situations, underscoring the need for appropriate behavioral responses.^(16)^

Diverse nonhuman animals engage in cooperative behaviors, including those influenced by empathy, as a fundamental aspect of their coexistence. This is based on the ability to differentiate self-generated stimuli from external stimuli produced by other organisms as well as to understand how each entity perceives its body within its surroundings.^(16,17)^

While initially considered an innate and individual characteristic, research suggests that empathy is malleable and susceptible to development through various approaches, even beyond standardized tools.^(18,19)^ This finding holds particular significance for human-animal interactions, where specific strategies can be employed to facilitate empathic behaviors. Offering knowledge and experiences that resonate emotionally and stimulate imagination is a promising intervention.^(20,21)^

Empathic interactions between humans and other animals, particularly in Brazil, remain relatively unexplored. Concerns regarding animal welfare have existed for some time, driven by various factors, including commercial pressures,^(22)^ scientific advancements,^(23)^ and food safety concerns,^(24)^ however, specific discussions on empathy towards animals emerged only around 40 years ago.^(25)^ It was not until the early 21^st^ century that this topic gained significant attention and recognition, coinciding with the growing awareness of its crucial role in environmental conservation policies.^(26,27)^

The Empathy Scale focused on Groups (ESG) was validated in Brazil in 2010. The ESG is designed to evaluate empathy towards specific minority groups and their experiences of suffering arising from social circumstances. While the scale can be employed to gauge empathy towards other animals, questions specifically addressing animals are limited and cannot be administered independently.^(28)^

In 2013, a study from Hong Kong introduced an instrument called Dispositional Empathy with Nature (DEN), which intended to reiterate the concept of empathic disposition rather than empathic induction. However, this scale does not directly encompass human perceptions of animals.^(29)^

The Animal Empathy Scale (AES)^(26)^ emerged from the assessment of empathy in undergraduate veterinary students in the UK, with the aim of identifying potential differences in empathy levels based on gender and year of study. Subsequently, in 2016, this instrument was adapted for the Portuguese population^(30)^ becoming the only tool described in the literature explicitly designed to measure human-animal empathic relationships.

Considering the global scarcity of instruments to assess human-animal empathy, coupled with the specific need for such a tool in Brazil, this study presents a culturally adapted and validated version of the AES for Brazilian audiences.

## OBJECTIVE

This study describes the adaptation of the Portuguese version of the Animal Empathy Scale to the Brazilian context and reports the validation assessments of the Brazilian version, designated AES-Brazilian version.

## METHODS

This study entails the cross-cultural adaptation and validation of the AES, derived from the broader "*Um tempo com e-Natureza*" (A time with e-Nature) project. This project, which was funded by the Boticário Group Foundation for Nature Protection, aimed to evaluate a nature-based intervention model designed to enhance well-being, connection, and engagement with nature, among other outcomes. Data were collected between April and June 2022 by the expert committee and between October 2022 and February 2023 by other research participants.

The AES originated in Scotland in 2000 and involved university students from Veterinary and Psychology courses. Initially comprising 28 items^(26)^ the scale was refined to include 22 items.^(31)^ Throughout its iterations, the AES has consistently employed a Likert scale with nine possible responses, ranging from "I strongly agree" to "I strongly disagree".^(26,31)^

Originally, AES assertions were grounded in the Questionnaire Measure of Emotional Empathy (QMEE),^(32)^ designed to evaluate emotional or affective empathy. Consequently, the AES appears to assess empathy as a unifactorial construct, as its publication lacks a description of factorial analyses and includes only a reliability assessment, yielding a Cronbach's alpha value of 0.78.^(31)^

During its adaptation for Portugal, 13 statements were retained from the original 22 and subsequently categorized into two subscales: Emotional Attachment with Animals (EAA) and Empathic Concern with Animals (ECA). This categorization was followed by exploratory and confirmatory factor analyses. Importantly, a positive correlation between these subscales was sustained (*r*=0.58), and the scale exhibited internal consistency and reliability, as evidenced by a Cronbach's alpha value of 0.84.^(30)^ The nine possible responses, ranging from 1 to 9, are as follows: 1 = strongly disagree, 2 = strongly disagree, 3 = disagree, 4 = slightly disagree, 5 = neither agree nor disagree/don't know, 6 = slightly agree, 7 = agree, 8 = agree a lot, and 9 = agree very much.

We started with the Portuguese version,^(30)^ benefiting from its linguistic similarity, and established construct validity analyses. The use of the original scale^(31)^ in the current study was authorized by the author.

To assess the validity of the Brazilian version of the AES (AES-Brazilian), we employed three validity categories,^(33)^ described in the following sections: content evaluation by an expert committee, internal structure, and relationships with external measures.

### Content evaluation by an expert committee

The expert committee comprised seven judges selected based on specific criteria: professional experience and/or holding a PhD in fields relevant to nature, zoology, veterinary science, or interpersonal relationships, and familiarity with Portuguese culture. The specialists received both a Portuguese version of the scale and a version containing the suggested changes. The judges were asked to indicate their agreement with the proposed modifications. If they partially agreed or disagreed, they were asked to justify or suggest improvements.

Through two rounds of evaluation, we achieved a minimum agreement of 80% for each statement as determined by the Content Validity Index (CVI).^(34)^ Subsequently, the scale was validated and made available to the study participants.

### Internal structure

Exploratory Factor Analysis (EFA), Confirmatory Factor Analysis (CFA), Cronbach's alpha, and McDonald's omega were used to assess the internal structure of the AES.

### Relationships with external measures

Convergent validity was assessed to determine the degree to which the scale correlated with another instrument that measured a related construct. We examined the correlations between the AES subscales and the Santa Clara Brief Compassion Scale using Spearman's correlation coefficients. Originating at the University of Santa Clara, California, United States, the Santa Clara Brief Compassion Scale was developed in 2005 to extend the concept of compassion beyond the religious context.^(35)^ It is a Likert-type scale consisting of five items scored from 1 (not true for me) to 7 (very true for me) and was validated for use in Brazil in 2018.^(36)^

Following content validation, the scale was administered to study participants who were over 18 years old and frequented five local natural areas in the State of São Paulo. A total of 386 participants completed all the items of the instrument. Questionnaires were completed on the Redcap^®^ institutional platform after evaluation and approval by the Research Ethics Committee of the *Hospital Israelita* *Albert Einstein* (CAAE: 54479721.0.0000.0071; #5.872.086). In adherence to national and international legal requirements, all participants provided informed consent by signing the Term of Free and Informed Consent (TCLE) before completing the questionnaires.

Subscale comparisons among groups of interest were performed using Student's *t*-tests or one-way ANOVA according to the number of groups, as normal distribution assumptions were not rejected for the subscales. Results were presented as means and standard deviations by group, with corresponding p-values indicating differences. All analyses were performed using the R packages^(37)^ psych,^(38)^ lavaan,^(39)^ and semPlot ^(40)^ with a significance level of 0.05.

## RESULTS

Seven judges, comprising five women and two men with ages ranging from 33 to 63 years participated in the content evaluation. Professionally, two were biologists, one occupational therapist, one nurse, one geologist, one forestry engineer, and one naturalist. Five judges resided in São Paulo, Brazil, and two were based in Porto, Portugal.

During the first round of evaluation, one statement and nine assertions achieved a minimum agreement of 80%. In the second round, the remaining four assertions attained a Content Validity Index (CVI) of 1. Table 1 presents the CVIs for each assertion in each round.

**Table 1:** Content Validity Index per item, statement, and assertive in each round of judges’ evaluation

AES item	CVI 1^st^round	CVI 2^st^round
Statement	1	-
Assertive 1	1	-
Assertive 2	0.85	-
Assertive 3	1	-
Assertive 4	0.71	1
Assertive 5	1	-
Assertive 6	0,71	1
Assertive 7	1	-
Assertive 8	0.28	1
Assertive 9	1	-
Assertive 10	0.57	1
Assertive 11	0.85	-
Assertive 12	1	-
Assertive 13	0.85	-

AES: Animal Empathy Scale; CVI: Content Validity Index.

In the second evaluation by the judges, two individuals did not respond to the requests and therefore did not evaluate the items. Consequently, the content validation process was concluded with the full participation of five experts.

A total of 386 participants were enrolled in the study. They included 59% women, 40% men, and 1% non-binary individuals. The mean age of the participants was 42 years (SD=14 years), with ages ranging from 18 to 82 years. A total of 83% of the respondents had pets during childhood, whereas 66% had pets at the time of participating in the survey.

For the EFA, 260 responses were selected from a total of 386, ensuring a minimum of 20 responses for each item on the scale. The adequacy of the sample was assessed using Bartlett's sphericity test, the Measure of Sample Adequacy (MSA), and the Kaiser-Meyer-Olkin index (KMO). Bartlett's test yielded a *p<0.001,* indicating adequacy. The KMO value was 0.90, and the MSA for each item was >0.80, indicating good adequacy.

The EFA was conducted using the ordinary least squares method and oblimin rotation, resulting in a two-factor solution that jointly explained 54% of the total variability of the items. All factor loadings and communalities were >0.4, as shown in table 2. Notably, the two factors in this solution exhibited a negative correlation.

**Table 2:** Exploratory factor analysis results: factor loadings and communalities obtained for the two subscales

Item	Assertive	ECA	EAA	Communalities
I03	I get apprehensive when I see an elderly and helpless animal	0.82	0.07	0.63
I09	I get apprehensive when I see an animal suffering	0.79	-0.06	0.67
I01	I feel sad when I see an animal alone in a cage	0.73	-0.01	0.55
I13	I hate seeing birds locked up in cages where they don't even have room to fly	0.71	0.11	0.45
I12	I would always try to help if I saw a lost dog	0.69	-0.02	0.49
I05	I get angry when I see animals being mistreated	0.68	-0.07	0.51
I07	My pet(s) has/have a great influence on my mood	0.61	-0.10	0.43
I08	Sometimes I am surprised by the intensity of sadness that some people feel when their old pet dies	0.10	0.80	0.59
I04	There are many people who are overly affectionate with their pets	0.19	0.75	0.49
I10	People usually exaggerate the emotions and feelings they believe animals have	-0.16	0.72	0.63
I06	It's silly to be overly fond of a pet	-0.13	0.70	0.58
I02	I feel uncomfortable when I see people pet or kiss their pets in public	-0.26	0.59	0.53
I11	I find it annoying when dogs jump on top of me and lick me to greet me	-0.39	0.40	0.44
% variance		32	54	

ECA: Empathic Concern with Animals subscale (items I03, I09, I01, I13, I12, I05, I07); EAA: Emotional Attachment with Animals subscale (items I08, I04, I10, I06, I02, I11).

**Figure 1:**
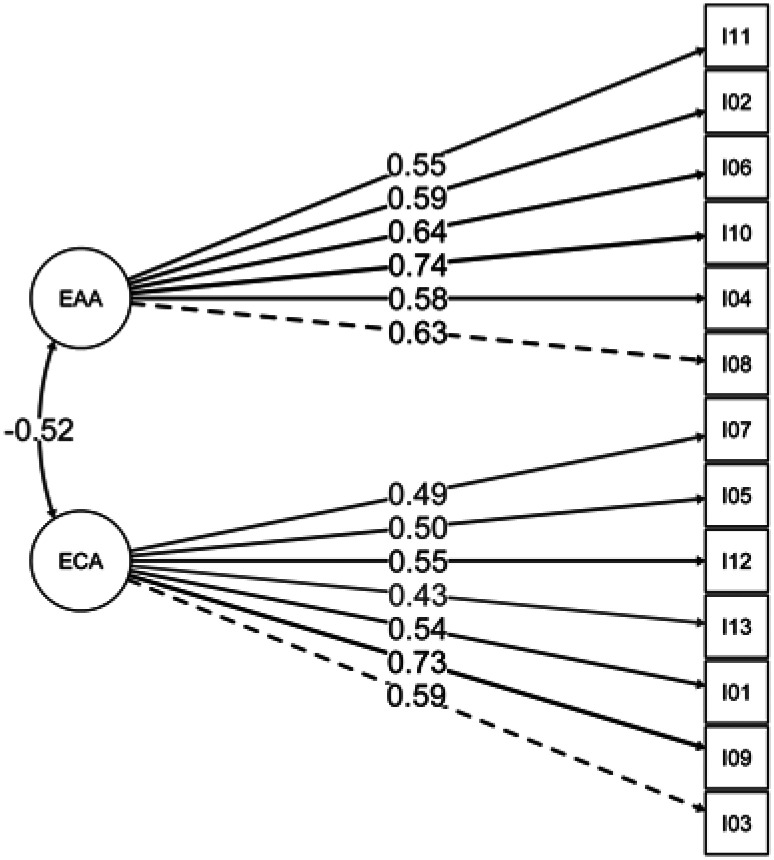
Path diagram of the confirmatory factor analysis applied to all samples

The 126 remaining samples were subsequently subjected to CFA using the maximum-likelihood technique. Model fit was assessed using absolute fit indices: χ^2^/degrees of freedom ratio (χ²/df) of 1.79, indicating excellent fit (114.5/64); standardized root mean square residual (SRMR) of 0.079, suggesting good fit; root mean square error of approximation (RMSEA) of 0.079, confirming acceptable fit; and Tucker-Lewis index (TLI) of 0.847 and comparative fit index (CFI) of 0.814, both indicating satisfactory fit.^(34)^ Based on these indices, a two-factor model (based on the two subscales), EAA and ECA, adapted from the Portuguese version, was retained. Furthermore, CFA was conducted on all the samples, as shown in figure 1.

The internal consistency of the EAA and ECA subscales was assessed using Cronbach's alpha and McDonald's omega coefficients. Both measures indicated good internal consistency: ECA yielded alpha and omega values of 0.75, whereas EAA yielded 0.79 for both coefficients. The combined analysis of all items yielded an alpha of 0.82 and an omega of 0.84. As values between 0.7 and 0.8 are considered acceptable,^(41)^ these results demonstrate satisfactory internal consistency for both subscales.

As presented in table 1, the ECA subscale is composed of the following seven items: I01-I feel sad when I see an animal alone in a cage; I03-I get apprehensive when I see an elderly and helpless animal; I05-I get angry when I see animals being mistreated; I07-My pet(s) has/have a great influence on my mood; I09-I get apprehensive when I see an animal suffering; I12-I would always try to help if I saw a lost dog; and I13-I hate to see birds locked up in cages where they don't even have room to fly. Individual scores for each item can take discrete values from 1 to 9. To derive the final ECA score, individual scores were averaged, resulting in a value ranging from 1 to 9.

The EAA subscale, as presented in table 1, is composed of the following six items: I02-I feel uncomfortable when I see people petting or kissing their pets in public; I04-There are many people who are overly affectionate with their pets; I06-It's silly to be overly fond of a pet; I08-Sometimes I am surprised by the intensity of sadness that some people feel when their old pet dies; I10-People usually exaggerate the emotions and feelings they believe animals have; I11-I find it annoying when dogs jump on top of me and lick me to greet me. Individual scores for each item can take discrete values from 1 to 9. It is important to note that the EAA items exhibit a *negative connotation*, wherein a higher attributed score indicates a lower emotional attachment. Consequently, the scores for each item were inverted by subtracting the actual score from 10 (*inverted* *score = 10 - actual score*). Similar to ECA, the final EAA score was derived by averaging the individual inverted scores. This approach ensured that both the ECA and EAA scores range from 1 to 9.

**Figure 2:**
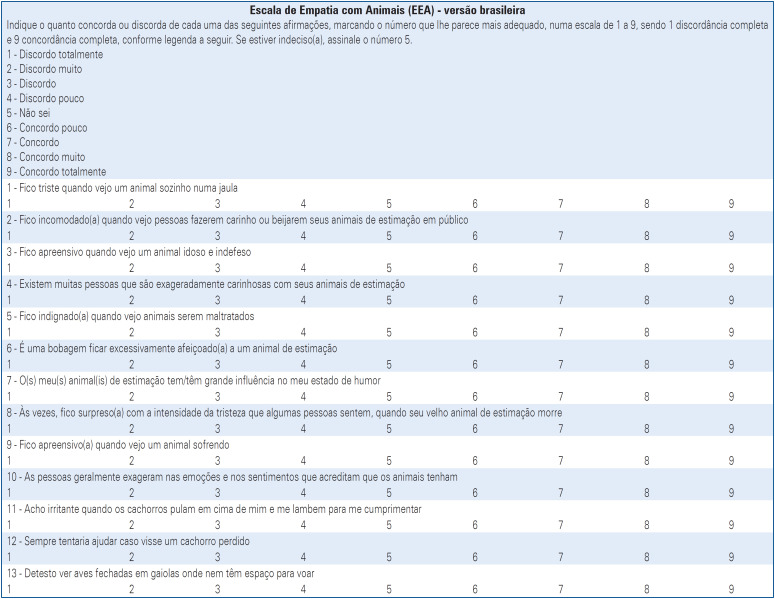
Brazilian version of the Animal Empathy Scale (AES-Brazilian version)

Source: Adapted from Emauz A, Gaspar A, Esteves F, Carvalhosa SF. Adaptação da Escala de Empatia com Animais (EEA) para a população portuguesa. Análise Psicol. 2016;2(34):189-201.(30)

The final ready-to-use AES-Bazilian version is shown in figure 2. It consisted of 13 items. Seven items assessed ECA, and six items assessed EAA. For each item, participants indicated their level of agreement or disagreement on a scale of 1 to 9, where 1 represented "strongly disagree" and 9 denoted "strongly agree."

We conducted score calculations for the sample of 386 individuals, resulting in a range of 2.9 to 9.0 for ECA, with a mean of 7.8 and SD of 1.0. For EAA, the score range was between 2.0 and 9.0, with a mean of 6.5 and SD of 1.7.

Upon stratifying the participants by sex and age, we observed higher mean score values, indicating a stronger emotional connection with animals, among female and non-binary participants, as well as, in individuals up to 40 years of age. Additionally, we found evidence of greater empathic concern toward animals among those who reported having pets during childhood. Furthermore, we noted that current pet ownership was positively associated with both ECA and EAA. Table 3 presents the relationship between ECA and EAA scores and age, sex, and pet ownership status.

**Table 3:** Relationship between Animal Empathy Scale scores and age, gender, and pet ownership status

		Empathic concern with animals	Emotional attachment to animals
		Mean (SD)	Mean (SD)
Age (years)			
	Up to 40, n=174	7.8 (1.0)	6.8 (1.6)
	41 - 60, n=171	7.7 (1.1)	6.3 (1.7)
	61 or older, n=38	7.9 (0.8)	6.2 (1.8)
	p value	0.700*	0.005*
Gender			
	Male, n=154	7.6 (1.0)	6.1 (1.8)
	Female, n=228	7.8 (1.0)	6.7 (1.6)
	Non-binary, n=4	8.0 (0.8)	6.7 (1.2)
	p value	0.100*	0.002*
Had a pet in childhood			
	Yes, n=319	7.8 (1.0)	6.5 (1.7)
	No, n=66	7.5 (1.0)	6.4 (1.8)
	p value	0.019^#^	0.600^#^
Has a pet now			
	Yes, n=256	7.9 (0.9)	6.7 (1.7)
	No, n=129	7.4 (1.1)	6.0 (1.7)
	p value	<0.001^#^	<0.001^#^

* One-way ANOVA; ^#^ Student *t*-test comparison.

We assessed the convergent validity of the AES-Bazilian version by measuring the Santa Clara Brief Compassion Scale scores. The results ranged from 1 to 7, with a mean of 5.9 and SD of 1.1. We subsequently evaluated the relationship between animal empathy scores and Santa Clara Brief Compassion Scale scores. The Spearman's correlation coefficient between ECA and compassion scores was 0.347, indicating a weak positive correlation. For EAA, Spearman's correlation coefficient with the compassion score was 0.057, representing a very weak (negligible) correlation.

## DISCUSSION

This study explored the human-animal-environment relationship by validating the AES in Brazil. The Portuguese version of the AES served as the starting point, and 13 items were retained after expert evaluation by seven judges. Subsequently, the adapted scale was administered to 386 participants. The analysis confirmed the validity of the resulting Brazilian version of the AES (AES-Brazilian version), establishing it as the first Brazilian instrument specifically designed to assess human-animal empathic connections.

Unlike the original AES and its Portuguese adaptation, this study explicitly details the content validation process and presents the resulting CVI data. This distinguishes our approach and provides a clear starting point for further instrumental validity analysis. Notably, the Norwegian translation of the AES into their language involved a modified scoring method; however, no details regarding the adaptation process were reported.^(42)^

In our study, we achieved an excellent CVI score of 1 for 11 of the possible 14, exceeding the benchmark for satisfactory validity.^(43)^ This aligns with the positive results obtained in the Portuguese validation study, where EFA demonstrated good sample suitability with p<0.001 and a KMO value of 0.87.

In addition to the excellent CVI, we identified two factors (subscales or constructs): ECA and EAA. In the adapted Portuguese version, item 7 ("My pet(s) has/have a great influence on my mood") aligns more appropriately with the EAA subscale. However, in the Brazilian adaptation, this was included in the ECA subscale. In the Brazilian version, only negative statements were integrated into the EAA subscale, which rationalized the repositioning of Question 7 to the ECA. subscale. Furthermore, the capacity of pets to affect owners’ moods is well established. The attribution of human-like characteristics, anthropomorphizing tendencies, affection, and the experience of unconditional love towards pets contribute significantly to influencing tutors’ moods.^(44,45)^ Consequently, empathic relationships surpass emotional connections. Consequently, Question 7 was reclassified from EAA to the ECA subscale in the Brazilian version.

The AES-Brazilian version also presented high levels of internal consistency according to Cronbach's alpha (0.75 for ECA, 0.79 for EAA), aligning with the results of the Portuguese version (0.79 for ECA, 0.83 for EAA), and in the original version (0.78 for the entire questionnaire). The Portuguese and original versions did not mention the use of McDonald's omega. This coefficient was identified as a more accurate measure of reliability because it considered the factorial load of each item in the construct composition.^(46)^ In our analysis, the AES-Brazilian yielded a high McDonald's omega value (0.84 for the entire questionnaire), further solidifying its internal consistency and reliability.

Despite the excellent internal consistency of the AES-Brazilian version, concerns regarding its convergent validity remain. We foud weak correlations between the AES-Brazilian version and the Santa Clara Compassion Scale, mirroring previously reported low correlations between the Portuguese AES adaptation and the Interpersonal Reactivity Index (IRI) and between the original AES and the Questionnaire for the Measurement of Emotional Empathy (QMEE).^(31)^ These inconsistencies suggest the potential limitations of the chosen validation instruments. While seemingly suitable, these instruments may be inherently human-centric or may lack clarity when focusing on animal-directed relationships. This suggests that as opposed to empathy directed towards humans, empathy towards animals manifests through different pathways. Empathy directed towards other species often raises the concept of "approximate empathy," acknowledging the inherent limitations in fully understanding the emotional experiences of beings vastly different from humans.^(13)^ However, our cognitive abilities, combined with collective knowledge and personal experiences, empower us to predict animal emotions and cultivate a degree of empathetic connection. Some factors that influence our ability to empathize with other animals include gender, phylogenetic proximity, and living with pets.^(47)^ Anthropomorphizing, a strategy mediated by empathy, promotes conservation. Attributing feelings and emotions to animals can generate attitudes toward protection. In contrast, ignoring or disqualifying the possibility of animal sentience by distancing them from our empathetic concerns risks creating a "discharge" of care.^(48)^

We further observed an association between female sex and pet ownership with heightened emotional attachment. This trend was also noted among individuals aged up to 40 years, which is consistent with the findings of a study involving over 75,000 Americans. The highest levels of empathy were reported among middle-aged individuals, reflecting an inverted "U" pattern in empathy self-perception throughout life. This scenario may be associated with the incomplete cognitive development of younger people and the possible social and physical limitations experienced by older people.^(49)^

Some cultural barriers hinder empathetic attitudes towards animals, such as the use of the pronoun "it" instead of "he" or "she" in English, as well as the dissemination of stories, movies, or beliefs that attribute evil to certain species.^(13,27)^ However, despite these challenges, certain actions, such as recognizing animals’ individuality, history, and preferences, dedicating time to studying, caring for, or interacting with animals, increasing exposure to nature, and facilitating perspective-taking through reflection, storytelling, role-playing, and mimicking, can foster empathy.^(13)^ Moreover, developing reliable instruments to assess animal empathy is crucial, as it allows us to track the effectiveness of strategies intended to enhance it.

From the One Health perspective, fostering empathy extends beyond mere challenges and emerges as a key driver. Research suggests positive associations between empathy and the core values of One Health, including pro-conservation behaviors, effective communication, and collaborative teamwork.^(50,51)^ In the current context of climate change and environmental injustice, promoting empathy is essential for advancing equity among diverse stakeholders.^(50,51)^

In the post-pandemic world, reflections on the relationship between the environment and potential health issues have highlighted the significance of One Health boundaries in influencing decision-making and behavioral changes.^(50)^ At the core of One Health ethics lies the principles of equity, equivalence, and interspecies bonding, suggesting that empathy serves as the foundational step in fostering these connections in a healthy and respectful manner.^(52)^

It is imperative that we dedicate our empathetic capacity to all beings that inhabit our planet-humans, non-humans, or other forms of life. We must recognize the importance of our attitudes toward other humans and understand that our care must be comprehensive.

Not coincidentally, connection with nature mirrors the three pillars of empathy: affective, representing an individual's sense of care for the natural environment; cognitive, reflecting on how one feels connected; and behavioral, corresponding to the commitment to biodiversity conservation.^(7)^ Empathy entails feeling, thinking, and acting. Therefore, these three stages ar crucial for consolidating a connection with nature, allowing it to be regarded as an extension of the empathic process of natural spaces.

Studies have indicated that specific exercises for the development of empathy, particularly among adolescents and young people, can result in stronger connections and more conservative attitudes.^(53,54)^ These findings emphasize the factors that promote well-being and engagement^(8)^ and, most importantly, validate the expectations and potential of One Health, Planetary Health, and Eco Health.^(55)^ These approaches embody transdisciplinary strategies aimed at bridging the gap between individual needs and the global environmental care imperative. Moreover, the imperative of planetary care cannot be dissociated from the 17 Sustainable Development Goals (SDGs). Established in 2015, the SDGs aim to achieve eradication of poverty, comprehensive environmental protection, and a healthy and prosperous life by 2030.^(56)^ Following the COVID-19 crisis, the United Nations (UN) published a report highlighting threats to life in the Anthropocene. The report emphasized the essential function of global solidarity in resuming progress towards achieving the SDGs.^(57)^ The report "Animal Welfare: Contributing to Sustainable Development"^(58)^ identified five relevant SDGs positively impacted by animal welfare: Zero Hunger and Sustainable Agriculture (SDG 2); Health and Well-Being (SDG 3); Decent Work and Economic Growth (SDG 8); Industry, Innovation and Infrastructure (SDG 9); and Responsible Consumption and Production (SDG 12). This highlights how addressing animal health directly influences both human well-being and broader planetary health.

Compassion, as a consequence of the empathetic process, was identified as the key to raising awareness and fostering action towards achieving the SDGs. Implementing these goals represents a crucial step towards the harmonious and healthy coexistence of all living beings on the planet.^(59)^ Therefore, empathy, both as a valued principle and a guiding behavior towards other species, can influence the human perspective on nature and act as a driver for improving the quality of life.

However, this study has a few limitations. These include the difficulty of comparing it to other validations using the same instrument or similar constructs, as these often employ fewer validity processes and categories. Furthermore, over time, the rigor of the validation process has increased, demanding more detailed explanations of the parameters, unlike in previous publications. However, these limitations have not prevented the use of the established instruments in large-scale studies.

We hope that the AES-Brazilian version will be widely applied in the future. This will serve as a tool for reflecting on human-animal relationships and guiding and monitoring the results of interventions aimed at improving the health of all species.

## CONCLUSION

Our findings provide evidence for the validity of the proposed AES-Brazilian version. This can be used as the first Brazilian instrument used to assess empathic relationships between humans and other animals.
